# Raynaud’s Phenomenon, Anticentromere Antibodies and Digital Necrosis (RACAND) Is a Distinct Clinical Syndrome From Systemic Sclerosis: A Case Report and Literature Review

**DOI:** 10.7759/cureus.90362

**Published:** 2025-08-18

**Authors:** Stela Hrkac, Josko Mitrovic, Majda Golob, Ivana Pitesa Kosutic, Lea Salamon

**Affiliations:** 1 Department of Clinical Immunology, Allergology and Rheumatology, Department of Internal Medicine, Dubrava University Hospital, Zagreb, HRV; 2 School of Medicine, University of Zagreb, Zagreb, HRV; 3 Faculty of Pharmacy and Biochemistry, University of Zagreb, Zagreb, HRV

**Keywords:** anti-centromere antibodies, digital ischemia, digital necrosis, racand syndrome, systemic sclerosis

## Abstract

The occurrence of digital necrosis and Raynaud’s phenomenon with anticentromere (ACA) antibodies has been recognized as a rare clinical entity coined RACAND syndrome. Although these features occur in systemic sclerosis (SSc), RACAND syndrome lacks other features characteristic of SSc, such as sclerodactyly, skin thickening or organ involvement. Reports of this syndrome are scarce throughout the literature, with only 10 described cases of RACAND syndrome, but several more reports of digital gangrene associated with ACA lacking SSc features. In this article, we present the case of an 80-year-old woman who developed digital gangrene, with Raynaud’s phenomenon and ACA in the absence of other signs and symptoms and no SSc features. The patient was diagnosed with RACAND syndrome and responded well to treatment with iloprost and moderate doses of prednisone. Additionally, a review of the available literature of similar cases is shown in order for clinicians to gain better insight of disease characteristics and possible treatment options, as there are no established treatment guidelines. The majority of patients were females of older age, mostly without medical history which would predispose them to a peripheral vasculopathy. Treatment including prostanoids might yield more promising results, however this requires further dedicated studies. We argue that RACAND syndrome is distinct from SSc and that the absence of other typical diagnostic features of SSc or other systemic autoimmune disease is necessary for diagnosis. Clinician awareness of this entity is needed in the treatment of digital necrosis, as well as further studies to determine the best treatment options.

## Introduction

Digital ischemia is known to be one of the common manifestations of systemic sclerosis (SSc), as well as Raynaud’s phenomenon, which is considered a hallmark of the disease [1]. Additionally, anticentromere (ACA) antibodies, are among those considered to be pathognomonic in SSc diagnosis [2]. However, the association of Raynaud’s phenomenon, presence of ACA and digital necrosis has been coined RACAND syndrome - a rare clinical entity, described very few times throughout the medical literature [3]. It is described as an entity possibly independent of SSc, lacking other features which are specific to SSc, such as sclerodactyly, skin thickening or internal organ involvement (such as lung, gastrointestinal, kidney or heart involvement) [2,3]. Absence of other factors possibly contributing to digital necrosis, such as peripheral arterial disease or diabetes, is also characteristic, based on other similar reports throughout the literature. Although RACAND syndrome and SSc share some similar features, the clinical course and affected patient profile differ and in order to determine diagnostic criteria and establish treatment guidelines, detailed case reports and studies are needed. Even though there are only 10 described cases of RACAND syndrome throughout the literature, there are several more reports of digital gangrene associated with ACA lacking SSc features, suggesting it might be underrecognized. The present article describes a case of an 80-year-old woman diagnosed with RACAND syndrome and relays a review of the available literature in order to raise awareness and aid clinicians in future diagnosis and treatment.

Parts of this case report have been presented as a congress abstract at the 14th Central European Congress of Rheumatology (CECR) on December 6th, 2024.

## Case presentation

**Figure 1:**
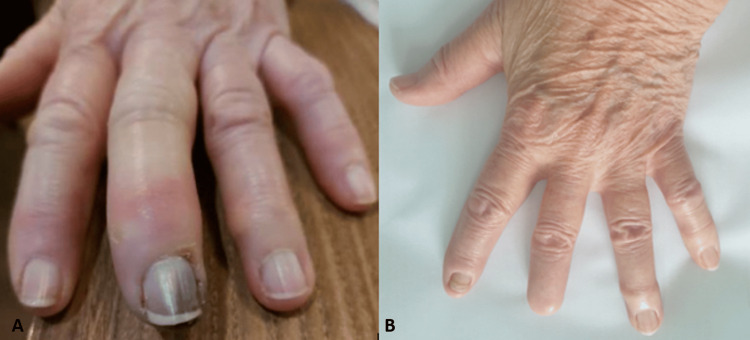
A. Initial phases of necrosis of the distal portion of the third fingertip of the left hand manifesting as discoloration and oedema of the skin, subcutaneous tissue and discoloration of the nailbed. B. The patient’s left hand after autoamputation of the distal phalanx of the third finger

**Figure 2:**
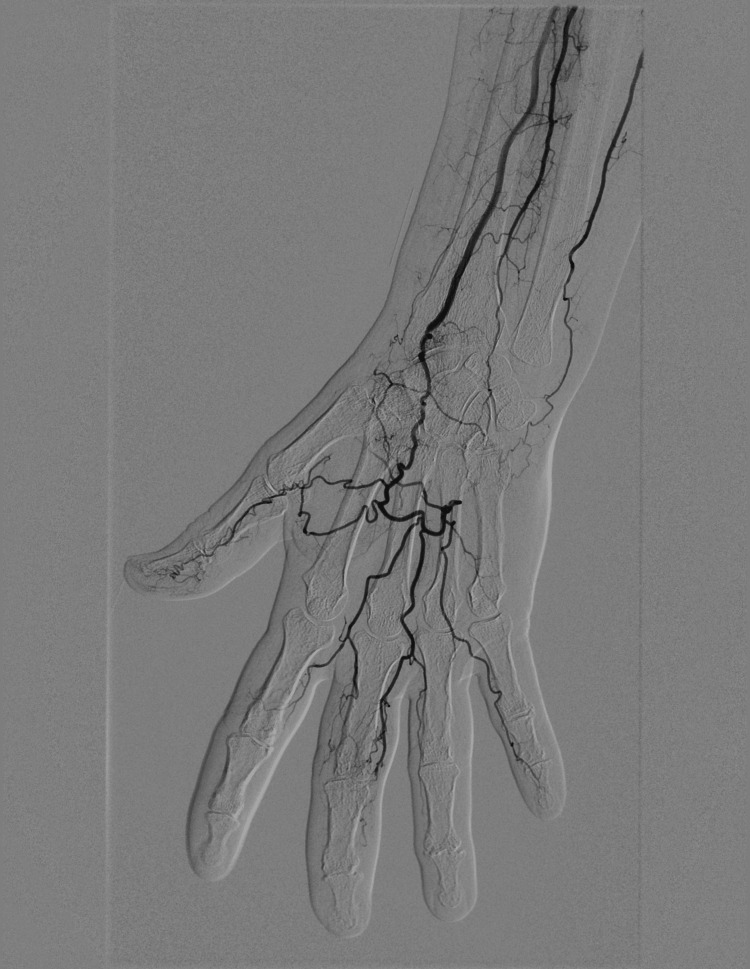
Digital subtraction angiography of the left distal upper extremity showed obliterative changes of the distal part of the ulnar artery and palmar arch with impaired distal flow.

An 80-year-old woman was referred to our Clinical Immunology, Allergology and Rheumatology department from the Division of Vascular and General Surgery after she presented with bilateral gangrene of the third fingertips. She reported swelling and erythema of the third fingertips bilaterally in the preceding two months, and dark discoloration (necrosis) starting from the nailbed two weeks prior (Figure 1A). One month following initial presentation, the right third gangrenous fingertip autoamputated, while cyanosis of the second fingertip bilaterally appeared. She had very mild Raynaud’s phenomenon (based on patient-reported symptoms and clinical assessment), without sclerodactyly, teleangiectasia or skin thickening. She had a medical history of hypertension and palpitations, and had otherwise been healthy and didn’t smoke. Initial laboratory findings showed elevated erythrocyte sedimentation rate (50mm/h) and C-reactive protein (56.6mg/L), no cytopenia, normal kidney function and lipid profile (Table 1). Inflammatory markers subsequently normalised and no clear signs of systemic infection, except a possible asymptomatic urinary tract infection, were found. Computed tomography (CT) angiography of the supraaortic arteries did not show any significant findings. Digital subtraction angiography of both hands revealed bilateral obliterative vasculopathic process (left hand shown in Figure 2, comparable finding of right hand not enclosed due to technical inadequacy of the image). Initial workup also identified atrial fibrillation (Afib), while transthoracic heart ultrasound showed no indirect signs of pulmonary artery hypertension or thrombi. Treatment was started with 10mg of amlodipine, 20mg of rosuvastatin, and 150mg twice a day of dabigatran (due to Afib) and hyperbaric oxygen therapy lasting approximately six weeks. After referral, additional workup showed positive antinuclear antibodies (ANA) with indirect immunofluorescence (IIF) testing with high titres of anticentromere antibodies (CENP-A and CENP-B), borderline positivity for anti-NOR 90, anti-fibrillarin, anti-Th/To and anti-RNA polymerase III. Testing for other specific antibodies was negative (anti-SSA, anti-SS-B, anti-Jo-1, anti-Sm, anti-Sm/RNP complex, anti-Scl-70, anti-PM-Scl 100 and 75, anti-PDGFR and anti-Ku). In addition to the findings listed in Table 1, the patient had negative cryoglobulins, antiphospholipid antibodies, hepatitis B, C and HIV serology, as well as normal serum protein electrophoresis. Multislice spiral computed tomography (MSCT) of the thorax, abdomen and pelvis showed no clear signs of malignant disease or interstitial lung disease. Capillaroscopy (using Lippolis optical video technology device) showed pallid-looking capillary loops (possibly due to vasospasm), but no clear capillary dropouts, giant capillaries or microhemorrhages. After reviewing similar cases throughout the literature, a diagnosis of RACAND syndrome was made (Table 2). Therapy with intravenous prostacyclin analog - iloprost (20µg daily in monthly five-day cycles) and 15mg of oral prednisone was started. After initiating iloprost therapy, cyanosis of the second fingertips regressed, no new signs of digital ischaemia appeared, while the left third gangrenous fingertip also autoamputated (Figure 1B). Due to good response to four monthly five-day cycles of iloprost, the drug dose was soon lowered to three-day cycles of 20µg every six weeks, and prednisone was gradually tapered to 5mg every other day. Additional diagnostic workup and ongoing follow-up (15 months after initial presentation) show no other signs of systemic or cutaneous manifestations of SSc, with the possible exception of oesophagitis, insufficiency of the gastric cardia and gastritis (which is managed well with proton pump inhibitors). The patient remains well, without any new signs of digital ischaemia or major complaints.

**Table 1:** Baseline laboratory findings WBC: white blood cell, CRP: C-reactive protein, ESR: erythrocyte sedimentation rate, ANA: antinuclear antibodies, anti-dsDNA: anti double stranded DNA antibodies, anti-CENP-A: anti Centromere Protein A antibody, anti-CENP-B: anti Centromere Protein B antibody, anti-MPO: anti myeloperoxidase antibodies, anti-PR3: anti proteinase 3 antibodies, RF: rheumatoid factor, IgG: immunoglobulin G, IgM: immunoglobulin M , IgA: immunoglobulin A, C3: concentration of  complement component 3, C4: concentration of  complement component 4

Variable	Patient value	Reference range
WBC count	10.9x10^9^/L	3.4 - 9.7x10^9^/L
‍Erythrocyte count	4.31 x10^9^/L	3.86-5.08 x10^9^/L
Hemoglobin	129 g/L	119-157 g/L
Platelet count	297 x10^9^/L	158-424x10^9^/L
CRP	56.6 mg/L	< 5.0 mg/L
ESR	50 mm/h	5-28 mm/h
ANA	Positive (1:320)	Negative (<1:80)
anti-dsDNA	3 IU/mL	<20 IU/mL
anti-CENP-A	+++ (highly positive)	- (negative)
anti -CENP-B	+++ (highly positive)	- (negative)
anti-MPO	1 U/mL	< 5 U/mL
anti-PR3	2 U/mL	< 5 U/mL
RF	11.5 IU/mL	<14 IU/mL
IgG level	14.3 g/L	7-16 g/L
IgM level	1 g/L	0.4-2.3 g/L
IgA level	2.2 g/L	0.9-4.5 g/L
C3	1.75 g/L	0.9-1.8 g/L
C4	0.29 g/L	0.1-0.4 g/L

**Table 2:** Summary of case reports describing digital gangrene associated with anticentromere antibodies without other systemic sclerosis (SSc) characteristics * only abstract available in English n/r – not reported, RP -Raynaud’s phenomenon, AH – arterial hypertension, WPW - Wolfe Parkinson White (WPW) syndrome, CHF – chronic heart failure, Afib - atrial fibrillation, CVA – cerebrovascular accident

Report	Sex (M/F)	Age (yrs)	RP	Affected limbs	Capillaroscopy findings	Comorbidities	Smoking	Treatment	Outcome	Development of SSc features (follow-up time)
Sachsenberg-Studer et al. (2000) [3]	F	43	+	fingers	giant capillaries	none	yes	iloprost	necrosis resolution	none (2 years)
	F	73	+	fingers	loop dropouts	AH	never	alprostadil	necrosis improvement	n/r
	F	84	+	fingers and toes	mild loop dropouts	AH	never	nifedipine, pentoxifylline, iloprost, aspirin, prednisone	necrosis reccurence, digital amputation	n/r
	F	86	+	fingers and toes	unremarkable	none	never	prednisone, iloprost, calcium channel blockers	necrosis resolution	n/r
Bolster et al. (2010) [4]	F	53	-	finger	unremarkable	WPW	never	clopidogrel, pentoxifylline, topical nitroglycerine, prednisone, antibiotic, nifedipine	necrosis resolution	none (follow -up time n/r)
Elqatni et al. (2014)* [5]	F	64	+	fingers and toes	n/r	none	never	prednisone, calcium channel blockers, iloprost	digital amputation	n/r
El Mahou et al. (2006) [6]	M	72	+	fingers and toes	enlarged microvascular loops, microhemorrhages	anaplastic small-cell bronchial carcinoma (found during workup)	never	aspirin, heparin, iloprost	digital amputation, necrosis recurrence	n/r
Abouzahir et al. (2010)* [7]	F	57	+	fingers and toes	n/r	primary biliary cirrhosis (found during workup)	n/r	iloprost, platelet aggregation inhibitors, calcium channel blockers	digital amputation	n/r
Takahashi et al. (1997) [8]	F	60	+	fingers and toes	n/r	n/r	n/r	n/r	n/r	n/r
	F	79	+	toes	n/r	CVA	n/r	n/r	n/r	n/r
	F	74	+	fingers	n/r	n/r	n/r	n/r	digital amputation	n/r
Grace et al. (2014) [9]	F	75	-	finger	n/r	none	never	n/r	n/r	n/r
Brown et al. (2001) [10]	F	87	possible	fingers	n/r	CHF	never	heparin, aspirin, antibiotics, nifedipine, iloprost	ischemia persistence	none (5 months)
Suman et al. (2025) [11]	F	60	-	finger	n/r	AH	never	thyroxine, statins, mupirocin, aspirin, sildenafil, telmisartan, nifedipine	digital amputation	n/r
Picillo et al (1998) [12]	F	39	+	fingers and toe	microaneurisms, capillary dilations	none	smoker	calcium channel blockers, aspirin, heparin, iloprost, prednisone, cyclophosphamide	digital amputation	n/r
Present case report	F	80	+	fingers	unremarkable	AH, Afib	never	amlodipine, statins, hyperbaric oxygen therapy, iloprost, prednisone	digital amputation, cyanosis resolution	none (15 months)

## Discussion

Digital ulcers and ischemia are common manifestations in SSc, considered the hallmark of SSc-related vasculopathy [1]. They occur in 30% of patients yearly and contribute to significant morbidity [1]. Raynaud’s phenomenon is also considered a hallmark of the disease and is essentially universal in SSc patients [2]. ANA patterns showing ACA, anti-topoisomerase and anti-reticulin antibodies are considered pathognomonic of SSc and are also included in the 2013 American College of Rheumatology/European League Against Rheumatism (ACR/EULAR) classification criteria for the disease [2]. However, in the case of our patient, digital necrosis, ischemia and ACA appeared in the absence of sclerodactyly and other diagnostic criteria of SSc. Similar cases, describing the association of ACA to digital ischemia, have been described throughout the medical literature and have been summarized in Table 2.

In this context, Takahashi et al. were the first to describe a series of six patients presenting with ulcers/ischemia of the extremities, Raynaud’s phenomenon (five/six cases) and positive ACA [8]. Other hallmark signs of SSc were absent in three of these patients (shown in Table 2) [8]. In the year 2000, Sachsenberg-Studer et al. described four similar cases, proposing that the triad of Raynaud’s phenomenon, ACA and digital necrosis should be identified as a distinct entity coined by the acronym “RACAND syndrome” [3]. Although only a few reports have used this acronym as a diagnosis [3,5-7], there are more cases that report digital necrosis associated with anticentromere antibodies without sclerodactyly or other features characteristic of systemic autoimmune disease (Table 2). Chatterjee et al. also described a patient diagnosed with RACAND syndrome, although a previous diagnosis of limited cutaneous systemic sclerosis (lcSSc) was present, and antiphospholipid antibodies were found [13].

When analysing similar cases throughout the literature, as shown in Table 2, it is observable that the patients were mostly female (15 of 16 analysed cases- 93.8%), of older age (mean age 67.9 years) and predominantly non-smokers without significant medical history that would predispose them to peripheral vasculopathy. Namely, none of the described patients had a history of diabetes or peripheral arterial disease. Unfortunately, most of the cases ended with surgical or autoamputation of the affected digits; however, reports of improvement or resolution of ischemia (including our own case) suggest that treatments including prostanoids and perhaps prednisone (among others) might yield more promising results.

Unlike SSc, treatment with other immunosuppressants and endothelin receptor inhibitors in patients with RACAND syndrome has not been studied.

We argue that for the diagnosis of RACAND syndrome, the absence of other typical diagnostic features of SSc (such as sclerodactyly and skin thickening) and other systemic autoimmune diseases is of importance, as it is an entity distinct from SSc [3]. Our patient, along with several others (Table 2), did not develop other SSc features over time; however, clinical features during long-term follow-up in this condition are underreported and need to be studied further. Other autoimmune conditions were excluded from differential diagnosis in our patient, such as small-vessel vasculitis or antiphospholipid syndrome, based on the absence of other clinical features or symptoms, as well as normal specific laboratory findings (e.g., negative ANCA and antiphospholipid antibodies). Furthermore, Sachsenberg-Studer et al. argue that ACA should be considered a risk factor for digital necrosis independent of cutaneous sclerosis (i.e., various subsets of SSc) as ACA could be responsible for arterial occlusion by enhancing platelet activation, endothelial damage and increased levels of vasoconstrictive mediators [3]. Takahashi et al. also suggested that ACA may be directly toxic to endothelial cells, with some authors even speaking of an “ACA-associated vasculitis” [8,12]. In more recent medical literature, anti-endothelial antibodies, including CENP-B, have been described as potential functional antibodies in SSc, associated with vascular involvement (digital vasculopathy) by activating endothelial cell modulation of fibroblast dysfunction and antibody-dependent cell-mediated cytotoxicity (ADCC) with endothelial apoptosis [2]. This might in part explain why in our case (along with several others, as shown in Table 2) typical Raynaud’s phenomenon can be absent, as it potentially plays a role only by aggravating the aforementioned ACA-mediated endothelial damage through vasospasm. The notion of “ACA-associated vasculitis” and ADCC in the pathogenesis of RACAND syndrome might provide a rationale for using glucocorticoids as part of the treatment protocol, particularly in cases (such as our patient) where inflammatory markers are elevated in the absence of infection. However, based on the current literature, there are no indications that high-dose glucocorticoids would have an obvious favourable effect and especially in elderly patients, side effects must be considered.

We believe that RACAND syndrome should be a consideration for clinicians in the differential diagnosis of digital necrosis, especially in females over the age of 65 with no history of diabetes or peripheral arterial disease. Based on the presented review of literature, we suggest that patients, in order to be diagnosed with RACAND syndrome, should fulfil the obligatory criteria of digital necrosis and positive ACA while Raynaud’s phenomenon further supports the diagnosis. Furthermore, these patients must not be eligible for the diagnosis of SSc or other systemic autoimmune rheumatic conditions.

However, even if we consider RACAND syndrome as a distinct entity, it must be kept in mind that studies have shown that Raynaud’s phenomenon in association with ACA may predict and even predate SSc by decades, which is why long-term follow-up of these patients is essential [3,14]. Further studies are needed to elucidate the pathophysiology of this condition and to ascertain optimal treatment regimens, to hopefully yield better patient outcomes.

## Conclusions

Herein we have presented the case of an 80-year-old woman with digital ischemia, mild Raynaud’s phenomenon and positive ACA, who was diagnosed with RACAND syndrome. Additionally, a broad review of the available literature on this topic is meant to aid clinicians in diagnosis and treatment of RACAND syndrome. Our case, in conjunction with characteristics from previous reports, highlights the importance of considering this rare entity and potential diagnostic and pathophysiological importance of ACA antibodies during diagnosis and treatment of patients with digital ischemia, even in the absence of other signs of systemic autoimmune rheumatic disease.

Further studies on this topic are needed in order to further elucidate its pathophysiology, establish diagnostic or classification criteria and to determine optimal therapeutic options.
